# The Kinase MAP4K1 Inhibits Cytosolic RNA-Induced Antiviral Signaling by Promoting Proteasomal Degradation of TBK1/IKKε

**DOI:** 10.1128/Spectrum.01458-21

**Published:** 2021-12-15

**Authors:** Tian-Sheng He, Jingping Huang, Tian Chen, Zhi Zhang, Kuntai Cai, Jingge Yu, Liang-Guo Xu

**Affiliations:** a College of Life Science, Jiangxi Normal University, Nanchang, China; b School of Basic Medicine, Gannan Medical University, Ganzhou, China; Regional Centre for Biotechnology

**Keywords:** MAP4K1, innate immunity, TBK1, IFN-I, cellular antiviral response

## Abstract

TANK-binding kinase 1 (TBK1)/IκB kinase-ε (IKKε) mediates robust production of type I interferons (IFN-I) and proinflammatory cytokines in response to acute viral infection. However, excessive or prolonged production of IFN-I is harmful and even fatal to the host by causing autoimmune disorders. In this study, we identified mitogen-activated protein kinase kinase kinase kinase 1 (MAP4K1) as a negative regulator in the RIG-I-like receptor (RLR) signaling pathway. MAP4K1, a member of Ste20-like serine/threonine kinases, was previously known as a prominent regulator in adaptive immunity by downregulating T-cell receptor (TCR) signaling and B-cell receptor (BCR) signaling. However, its role in regulating antiviral innate immune signaling is still unclear. This study reports an undiscovered role of MAP4K1, which inhibits RLR signaling by targeting TBK1/IKKε for proteasomal degradation via the ubiquitin ligase DTX4. We initially identify MAP4K1 as an interacting partner of TBK1 by yeast two-hybrid screens and subsequently investigate its function in RLR-mediated antiviral signaling pathways. Overexpression of MAP4K1 significantly inhibits RNA virus-triggered activation of IFN-β and the production of proinflammatory cytokines. Consistently, knockdown or knockout experiments show opposite effects. Furthermore, MAP4K1 promotes the degradation of TBK1/IKKε by K48-linked ubiquitination via DTX4. Knockdown of DTX4 abrogated the ubiquitination and degradation of TBK1/IKKε. Collectively, our results identify that MAP4K1 acts as a negative regulator in antiviral innate immunity by targeting TBK1/IKKε, discover a novel TBK1 inhibitor, and extend a novel functional role of MAP4K1 in immunity.

**IMPORTANCE** TANK-binding kinase 1 (TBK1)/IκB kinase-ε (IKKε) mediates robust production of type I interferons (IFN-I) and proinflammatory cytokines to restrict the spread of invading viruses. However, excessive or prolonged production of IFN-I is harmful to the host by causing autoimmune disorders. In this study, we identified that mitogen-activated protein kinase kinase kinase kinase 1 (MAP4K1) is a negative regulator in the RLR signaling pathway. Notably, MAP4K1 promotes the degradation of TBK1/IKKε by K48-linked ubiquitination via the ubiquitin ligase DTX4, leading to the negative regulation of the IFN signaling pathway. Previous studies showed that MAP4K1 has a pivotal function in adaptive immune responses. This study identifies that MAP4K1 also plays a vital role in innate immunity and outlines a novel mechanism by which the IFN signaling pathway is tightly controlled to avoid excessive inflammation. Our study documents a novel TBK1 inhibitor, which serves as a potential therapeutic target for autoimmune diseases, and elucidated a significant function for MAP4K1 linked to innate immunity in addition to subsequent adaptive immunity.

## INTRODUCTION

The innate immune system is an elaborate defense mechanism that evolved over tens of millions of years and resists the invasion of microbial pathogens, including bacteria and viruses. The detection of pathogen-associated molecular patterns occurs through pattern recognition receptors, including RLRs (1), Toll-like receptors (TLRs) (2), Nod-like receptors (NLRs) (3), and cytosolic sensors for DNA (4). After stimulation with RNA virus, RLRs can recognize viral RNAs by the C-terminal regulatory domain and subsequently trigger activation of the downstream transcription factors interferon regulatory factor 3/7 (IRF3/7) and NF-κB, which leads to the robust production of type I interferons (IFN-I) and proinflammatory cytokines, respectively (5). IFN-I further activate a series of signaling cascades promoting the expression of antiviral interferon-stimulated genes (ISGs) through the coordinated activation of multiple transcription factors, such as STAT1/2 and IRF9, and subsequently initiate adaptive immune responses (6). To avoid autoimmune disorders, the accurate regulation of IFN-I production plays a significant role in antiviral responses.

As a noncanonical IκB kinase (IKK) family member, TANK-binding kinase 1 (TBK1) is a ubiquitously expressed serine/threonine protein kinase that participates in signal transduction of multiple signaling pathways, including immune response, inflammation, autophagy, insulin signaling, cell proliferation, and growth (7). Because TBK1 serves as a node protein in IFN-I signaling, its role in innate immunity attracts fanatical research and has made remarkable progress. Following infection by an RNA virus, endosome membrane sensor TLR3 and cytoplasmic sensors, including RIG-I or MDA5 (8, 9), could recognize RNA and activate downstream signaling pathways by recruiting to the adaptor TRIF or MAVS (also known as VISA, IPS-1, and Cardif) (10–13), respectively (8, 14). When stimulated with a DNA virus, cytosolic DNA sensors IFI16, cGAS, ZBP1, and DDX41 recognize double-stranded DNA and initiate a series of signaling cascades via membrane-associated adaptor STING (15–18). All these different adaptors (TRIF, MAVS, or STING) activated by an RNA or DNA virus need TBK1 to promote the phosphorylation of IRF3/7 and trigger nuclear translocation for binding to IFN-β *cis*-elements, resulting in the expression of IFN-I or inflammatory cytokines (19, 20). Given the vital role of TBK1 in innate antiviral immunity, TBK1 inhibitors may play indispensable roles in regulating host antiviral responses and are especially considered as promising therapeutic targets for autoimmune diseases (21).

Mitogen-activated protein kinase kinase kinase kinase 1 (MAP4K1; also known as hematopoietic progenitor kinase 1 [HPK1]) is one member of a germinal center kinase family and is expressed in multiple cell types, such as innate immune cells (macrophages, neutrophils, mast cells, and dendritic cells) and lymphocytes (22, 23). Previous studies showed that MAP4K1 has a pivotal function in adaptive immune responses by inhibiting AP-1-, NFAT-, ERK-, and NF-κB-mediated gene transcription (24–27) and is a negative regulator of functions in murine dendritic cells by breaking antigen (Ag) presentation (28). However, the role of MAP4K1 in innate antiviral immunity is unclear. In this study, we identify MAP4K1 as a TBK1-interacting partner through yeast two-hybrid screens and elucidate the mechanism of MAP4K1 as a negative regulator of innate antiviral immunity. Our results demonstrate that MAP4K1 promotes degradation of TBK1/IKKε by increasing K48-associated polyubiquitination via the ubiquitin ligase DTX4. Therefore, this study documents a novel TBK1 inhibitor, which serves as a potential therapeutic target for autoimmune diseases, and elucidated a significant function for MAP4K1 linked to innate immunity and subsequent adaptive immunity.

## RESULTS

### MAP4K1 negatively regulates the virus-triggered RLR signaling pathway.

**FIG 1:**
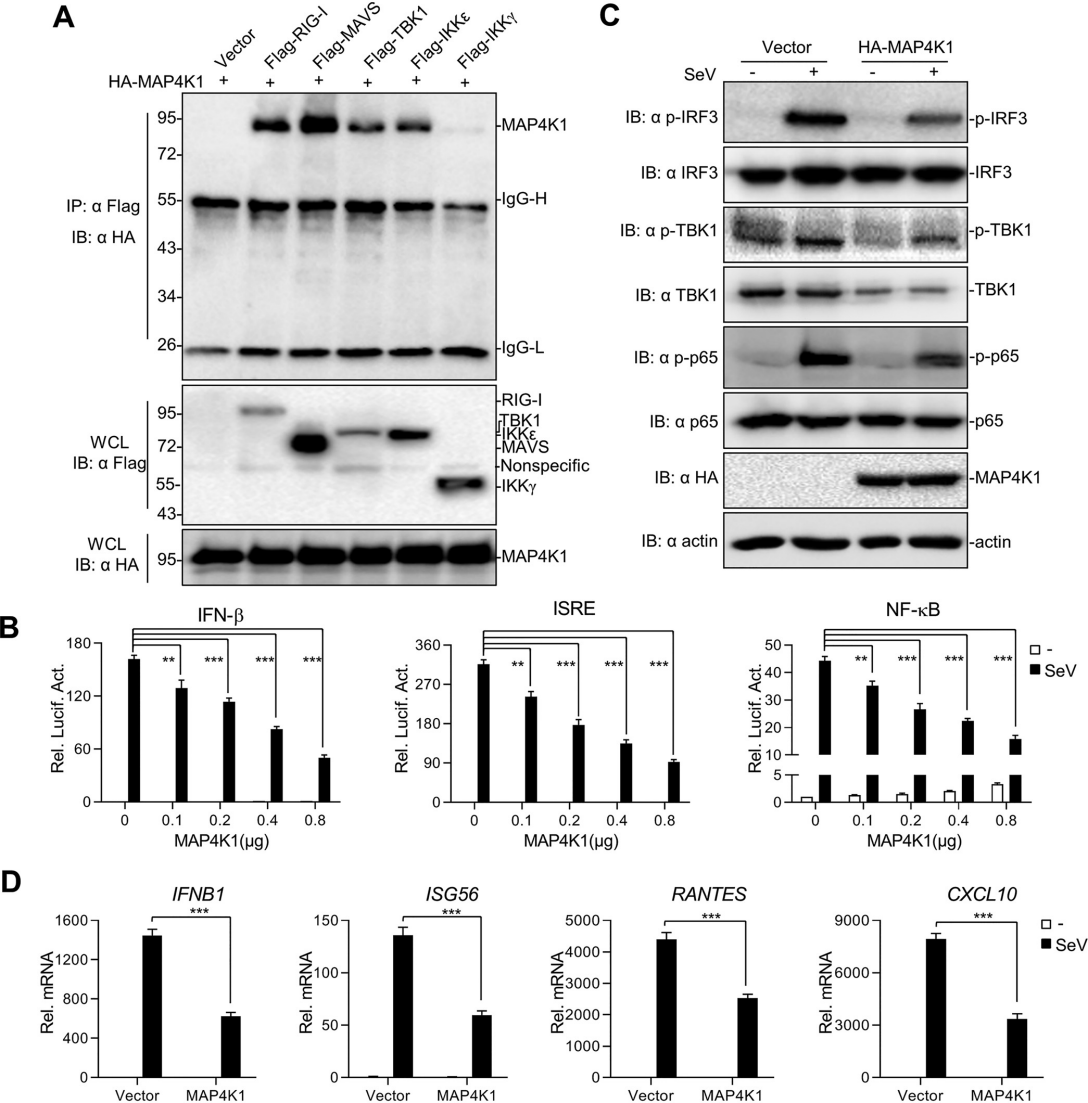
MAP4K1 negatively regulates the virus-triggered RLR signaling pathway. (A) MAP4K1 participates in the RLR signaling pathway. 293T cells (∼4 × 10^6^) were seeded in 100-mm dishes and transfected with various combinations (above lanes) of plasmids (8 μg each). Lysates were immunoprecipitated with anti-Flag and analyzed by immunoblotting with the indicated antibodies. (B) 293T cells (∼2.5 × 10^5^) were seeded in 24-well plates and transfected with plasmids carrying an IFN-β promoter, ISRE, or NF-κB luciferase reporter gene (100 ng/well) and pRL-TK (50 ng/well) as well as increasing amounts of an expression vector for MAP4K1. A luciferase assay was performed after treatment with SeV or not for 10 h; Con, control; Rel. Luc. Act., relative luciferase activity; Rel. mRNA, relative mRNA. (C, D) 293T cells (∼1 × 10^6^) were seeded in 6-well plates and transfected with vector or MAP4K1. Twelve hours after transfection, cells were treated with SeV or not for 10 h. Cell lysates were analyzed by Western blotting with the indicated antibodies (C), and mRNA levels were analyzed by real-time qPCR (D). Error bars indicate SEM; *, *P < *0.05; **, *P < *0.01; ***, *P* < 0.001; ns, no significant difference.

As a node protein regulating multiple signaling pathways, TBK1 plays a critical role in various immunopathological events, especially in innate immune responses to bacterial and viral infections. To identify new molecules tightly regulating the activity of TBK1, we searched a cDNA library for novel interacting partners of full-length TBK1 (as a bait protein) by a large-scale yeast two-hybrid screen system. As a result, we identified two novel proteins interacting with TBK1, THO complex subunit 7 homolog (THOC7) and MAP4K1 (GenBank accession number XM_011526403). We reported that THOC7 negatively regulates cellular antiviral responses by targeting TBK1. MAP4K1, which previously was known as a pivotal regulator in adaptive immune responses, may also play a significant role in antiviral innate immunity. To avoid false-positive clones in the yeast two-hybrid screen, further coimmunoprecipitation was performed to examine if MAP4K1 participates in the RLR antiviral signaling pathway. The results indicated that MAP4K1 indeed interacts with TBK1 and other RLR signaling pathway components, such as RIG-I, MAVS, and IKKε (Fig. 1A). Numerous reports have demonstrated that MAP4K1 has pivotal roles in various cellular events, particularly in T cell receptor (TCR)/B cell receptor (BCR) signaling (29). This evidence revealed that MAP4K1 serves a broader role in immunity and may play a significant role in antiviral innate immunity.

To investigate the hypothesis, dual-luciferase reporter assays were used to identify the functions of MAP4K1 in the regulation of IFN-β activity. The data indicated that MAP4K1 overexpression inhibited the activation of the IFN-β promoter induced by Sendai virus (SeV) in a dose-dependent manner (Fig. 1B). The promoter region of IFN-β is composed of interferon-stimulated response elements (ISRE), which are activated by the IRF family only, and a positive regulatory *cis*-element (PRD), which is activated by NF-κB (30). To determine the mechanism of MAP4K1 regulating the activation of IFN-β, we further introduced two luciferase reporters (an ISRE luciferase reporter and an NF-κB luciferase reporter) in the experiments. The data showed that MAP4K1 overexpression also evidently attenuated the activation of transcription factors IRF3 and NF-κB induced by SeV (Fig. 1B).

Furthermore, overexpression of MAP4K1 also showed similar effects on polyinosinic:polycytidylic acid (poly I:C)-triggered activation of IFN-β and ISRE in similar experiments (Fig. S1A in the supplemental material). Interestingly, MAP4K1 could activate NF-κB without virus treatment, whereas it becomes an inhibitor of NF-κB in the stimulation by SeV. It has also been reported that MAP4K1 has different roles in NF-κB activation, where full-length MAP4K1 could activate IKKα/β, and the C-terminal fragment of MAP4K1 could suppress NF-κB activation (31). Consistent with those data, we assessed the phosphorylation of IRF3, TBK1, and p65 and found that overexpression of MAP4K1 significantly reduced the phosphorylation of endogenous IRF3, TBK1, and p65 triggered by SeV (Fig. 1C). Moreover, we analyzed levels of ISG mRNA by real-time quantitative PCR (qPCR) (Fig. 1D) and found that MAP4K1 overexpression also resulted in lower expression of IFB1, ISG56, RANTES, and CXCL10. The results suggested that MAP4K1 negatively regulates the SeV-triggered IFN-I signaling pathway.

### Deficiency of MAP4K1 potentiates IFN-β production and antiviral responses.

**FIG 2:**
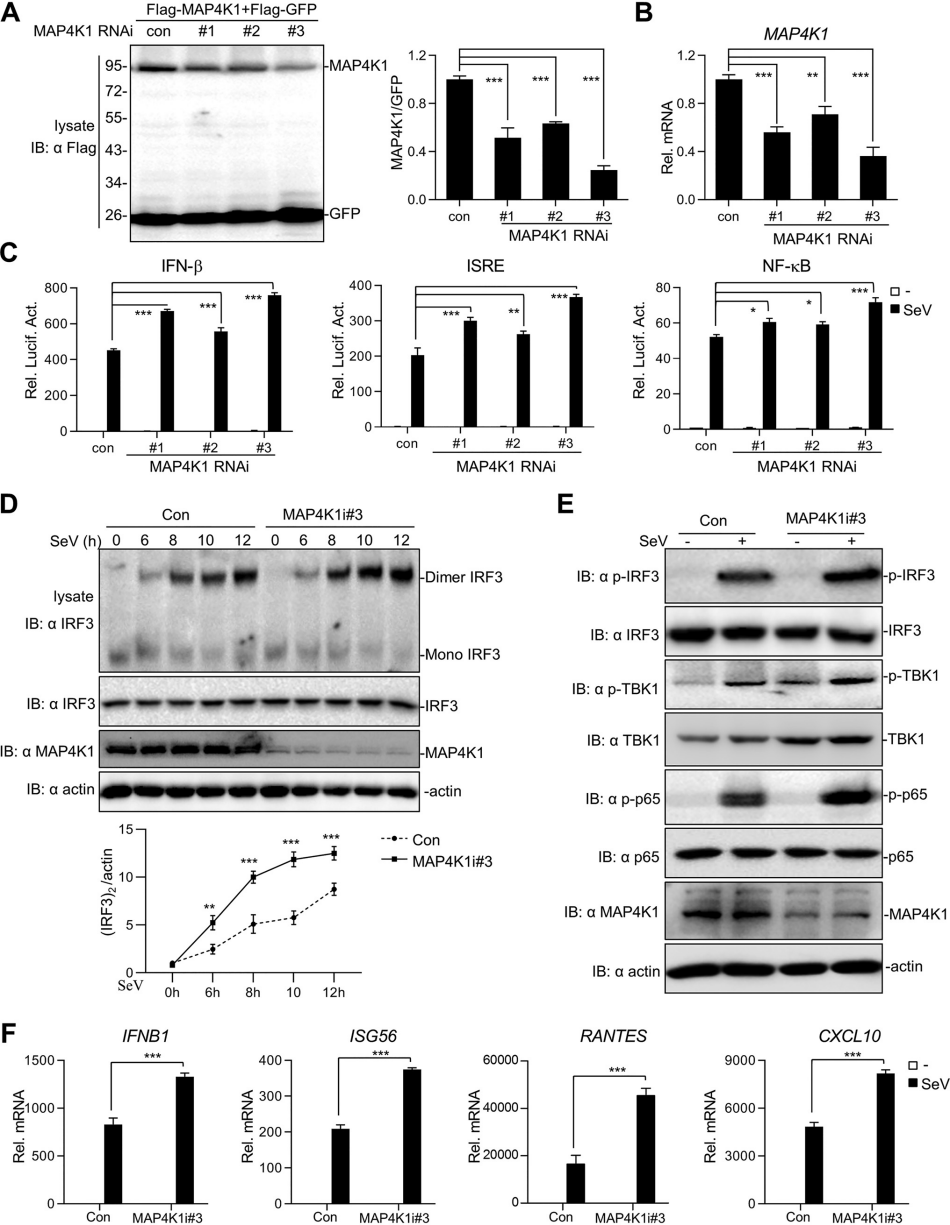
Knockdown of MAP4K1 enhances IFN-β and antiviral responses. (A, B) The effects of specific siRNAs suppressing MAP4K1 expression. 293T cells (∼1 × 10^6^) were either transfected with MAP4K1-specific siRNA (2 μg), Flag-MAP4K1 (1 μg), and Flag-green fluorescent protein (Flag-GFP) (0.2 μg) (A) or only transfected with MAP4K1-specific siRNAs (B). Twenty hours after transfection, cells were harvested and analyzed by Western blotting or qPCR. (C) Luciferase assays of 293T cells were performed by transfecting an IFN-β, ISRE, or NF-κB luciferase reporter with pRL-TK as well as MAP4K1 siRNA (0.5 μg/well). (D, E) An immunoassay of 293T cells was performed by transfecting MAP4K1-specific siRNA or control (3 μg), and cells were treated with SeV for indicated time points for dimerization assays by native PAGE (D) or for 10 h for immunoblotting with indicated antibodies (E). (F) Real-time qPCR assays of 293T cells transfected with MAP4K1-specific siRNA were performed by the method described in Fig. 1D. Error bars indicate SEM; *, *P < *0.05; **, *P < *0.01; ***, *P* < 0.001; ns, no significant difference.

To further probe the potential functions of endogenous MAP4K1 under physiological conditions, we next screened out the specific and effective small interfering RNA (siRNA) suppressing the expression of endogenous MAP4K1. According to the human MAP4K1 cDNA sequence, we constructed three siRNA expression plasmids (MAP4K1-RNAi 1 to 3) targeting MAP4K1 mRNA and assessed their efficiency by Western blotting and real-time qPCR. As a result, three siRNA constructs had different effects on the expression of MAP4K1. MAP4K1-RNAi 3 had the best knockdown efficiency on reducing the expression of MAP4K1 (Fig. 2A and B). MAP4K1-RNAi 1 and 2 had minor effects on reducing MAP4K1 expression, decreasing MAP4K1 levels to ∼50% and ∼70% of the control sample, respectively. Combined with the dual-luciferase reporter assay, we found that knockdown of MAP4K1 resulted in a higher degree of activation of the SeV-triggered IFN-β promoter, ISRE, and NF-κB (Fig. 2C) and poly I:C-triggered activation of IFN-β (Fig. S2A). Consistent with their effects on MAP4K1 expression, the degree of luciferase reporter was correlated with the efficiency of MAP4K1-specific siRNAs. MAP4K1-RNAi 3 resulted in the lowest expression of MAP4K1 and the highest activation of IFN-β luciferase reporter, so we selected this siRNA for the following experiments.

Because dimerization and phosphorylation of IRF3 are hallmarks of the activation of IFN-I, we further detected IRF3 activation by several approaches. Native PAGE showed that MAP4K1 knockdown observably enhanced the dimerization of endogenous IRF3 in different SeV treatment periods (Fig. 2D). Consistently, similar results were obtained by detecting the phosphorylation level of IRF3 in nucleoprotein extracts and found that knockdown of MAP4K1 enhanced poly I:C- or SeV-triggered phosphorylation of IRF3 (Fig. S2B). Furthermore, we found that knockdown of MAP4K1 also remarkably potentiated the SeV-induced phosphorylation of endogenous IRF3, TBK1, and p65 (Fig. 2E). In addition, knockdown of MAP4K1 resulted in a higher abundance of SeV-induced IFNB1, ISG56, RANTES, and CXCL10 (Fig. 2F). Therefore, these results revealed that knockdown of MAP4K1 potentiated IFN-I production.

**FIG 3:**
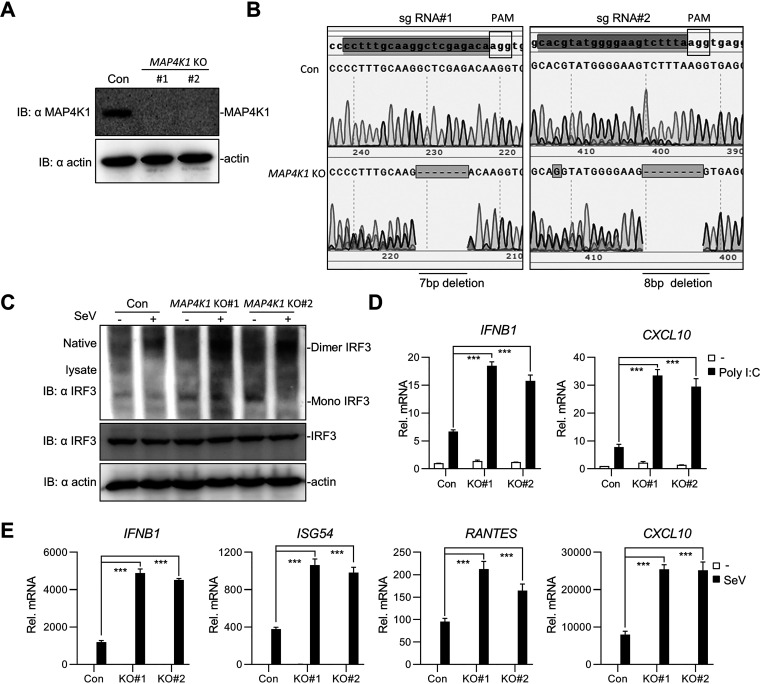
Knockout of MAP4K1 potentiates RLR-mediated signaling. (A, B) *MAP4K1*-knockout THP1 cells were generated by CRISPR-Cas9 technology. *MAP4K1*-knockout THP1 cells were analyzed by immunoblotting (A) and sequencing analysis (B). (C) *MAP4K1* knockout enhanced the dimerization of IRF3. *MAP4K1* knockout cells (2 × 10^6^) were uninfected or infected by SeV for 8 h and collected for dimerization assay by native PAGE. (D, E) Real-time qPCR assays of *MAP4K1*-knockout cells transfected with poly I:C (1 μg) (D) or infected with SeV for 8 h (E). Error bars indicate SEM; *, *P* < 0.05; **, *P* < 0.01; ***, *P* < 0.001; ns, no significant difference.

To further confirm the function of MAP4K1 in myeloid cells, we generated MAP4K1-deficient THP1 cells by clustered regularly interspaced short palindromic repeats (CRISPR)-Cas9 using a single guide RNA (sgRNA) targeting exon 1/2 of the *MAP4K1* gene. The single clones of *MAP4K1*-knockout cells were examined by Western blotting and sequencing analysis (Fig. 3A and B). Native PAGE showed that *MAP4K1* knockout markedly increased the dimerization of IRF3 induced by SeV (Fig. 3C). Furthermore, we found that *MAP4K1* knockout significantly enhanced the transcription of *IFNB1*, *CXCL10*, *ISG56*, and *RANTES* (Fig. 3D and E). Taken together, knockdown and knockout of *MAP4K1* expression potentiated IFN-β production and antiviral responses.

### MAP4K1 regulates the RLR signaling pathway by targeting TBK1/IKKε.

**FIG 4:**
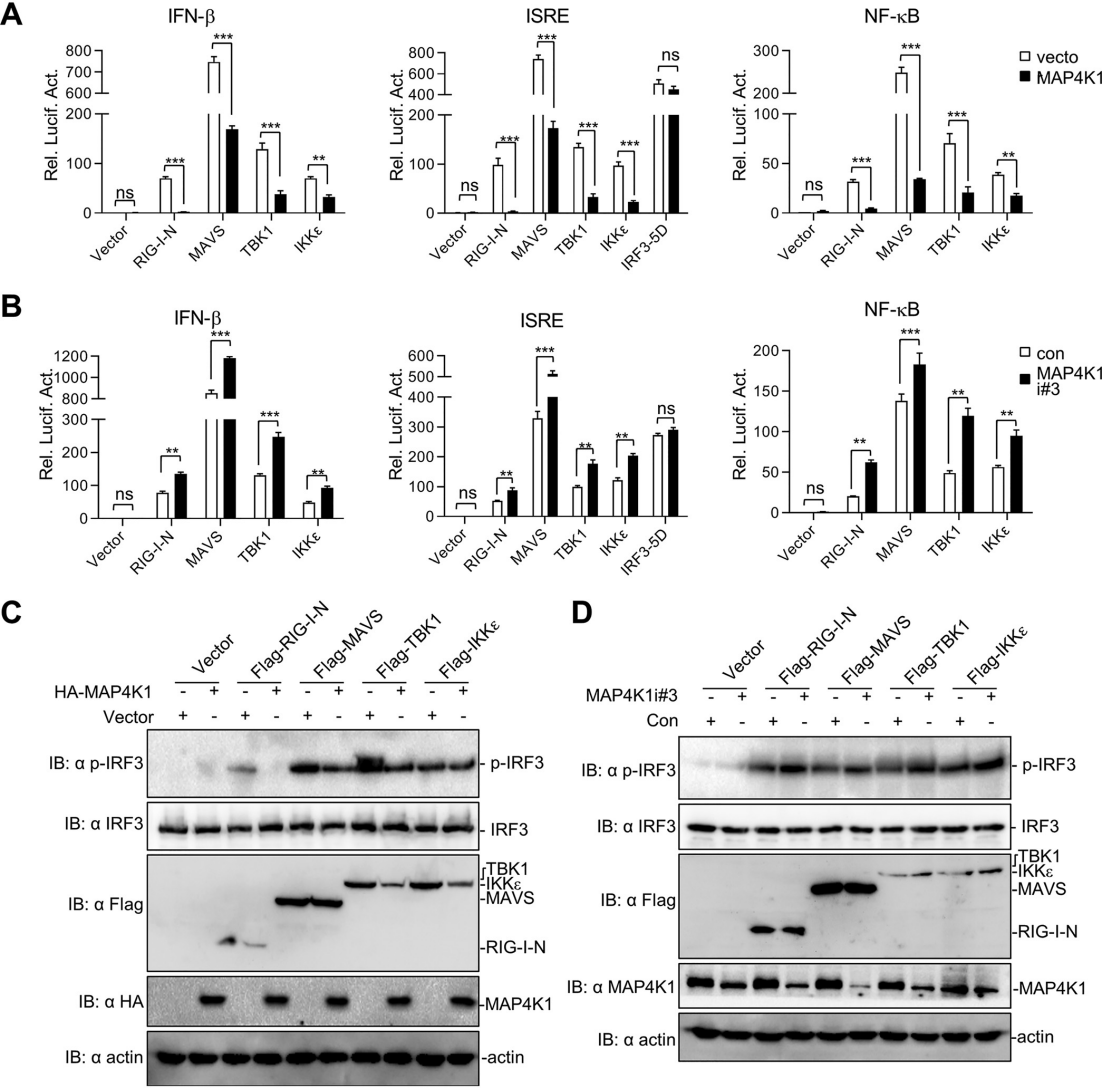
MAP4K1 regulates the RLR signaling pathway by targeting TBK1/IKKε. (A, B) 293T cells (∼2.5 × 10^5^) were transfected with a luciferase reporter gene carrying the IFN-β promoter, ISRE, or NF-κB (100 ng/well), pRL-TK (50 ng/well), RLR signaling molecules (0.5 μg) together with a MAP4K1 expression vector (0.5 μg) (A) or MAP4K1 siRNA constructs (0.5 μg) (B). Luciferase assays were performed 20 h after transfection. (C, D) 293T cells (∼1 × 10^6^) were transfected with RLR signaling molecules (2 μg) as indicated as well as a MAP4K1 expression vector (1 μg) (C) or MAP4K1 siRNA constructs (1 μg) (D). Cells were harvested and analyzed by immunoblotting with indicated antibodies after transfection for 20 h. Error bars indicate SEM; *, *P < *0.05; **, *P < *0.01; ***, *P < *0.001; ns, no significant difference.

To identify the potential targets of MAP4K1 in RLR-mediated signaling, we further introduced a node-downstream-activation model as previously described. The downstream signaling pathway was activated through transient transfection of RLR molecules, including RIG-I-N (containing only the RIG-I N-terminal CARD domain), MAVS, TBK1, IKKε, and IRF3-5D (a constitutively activated mutant of IRF3 by phosphate mimic). As shown in Fig. 4A, MAP4K1 overexpression reduced RIG-I-N-, MAVS-, TBK1-, and IKKε-mediated activation of ISRE, NF-κB, and IFN-β promoters in reporter assays. Consistently, MAP4K1 knockdown promoted RIG-I-N-, MAVS-, TBK1- and IKKε-mediated activation of ISRE, NF-κB, and IFN-β promoters (Fig. 4B). However, MAP4K1 overexpression or knockdown caused little effect on IRF3-5D-induced ISRE activation. Since RIG-I and MAVS function upstream whereas IRF3 functions downstream of TBK1/IKKε, we therefore inferred that MAP4K1 regulates RLR-mediated signaling upstream of IRF3 and targets TBK1/IKKε. Consistent with the results of reporter assays, MAP4K1 inhibited RIG-I-, MAVS-, TBK1-, and IKKε-mediated IRF3 phosphorylation (Fig. 4C), and MAP4K1 knockdown had consistent results on IRF3 phosphorylation (Fig. 4D). Collectively, these results strongly indicated that MAP4K1 regulated the RLR signaling pathway by targeting TBK1/IKKε.

### MAP4K1 promotes the degradation of TBK1/IKKε.

MAP4K1 is an essential serine/threonine protein kinase with various immune roles by interacting with several adaptors, including SLP-76 and BLNK. Following T-cell receptor (TCR) stimulation, MAP4K1 was activated by recruiting the adaptor SLP-76, which forms a signalosome complex. Then, the activated MAP4K1 phosphorylates SLP-76 and subsequently mediates the ubiquitination and degradation of SLP-76 by interacting with E3 ubiquitin ligase 14-3-3 (32, 33). Furthermore, a similar mechanism was found in B cell receptor signaling, which also phosphorylates the adaptor BLNK and induces the degradation of BLNK by ubiquitination (26). So, we hypothesized that MAP4K1 reduced the stability of the MAVS signalosome complex and induced proteasomal degradation of signaling molecules.

**FIG 5:**
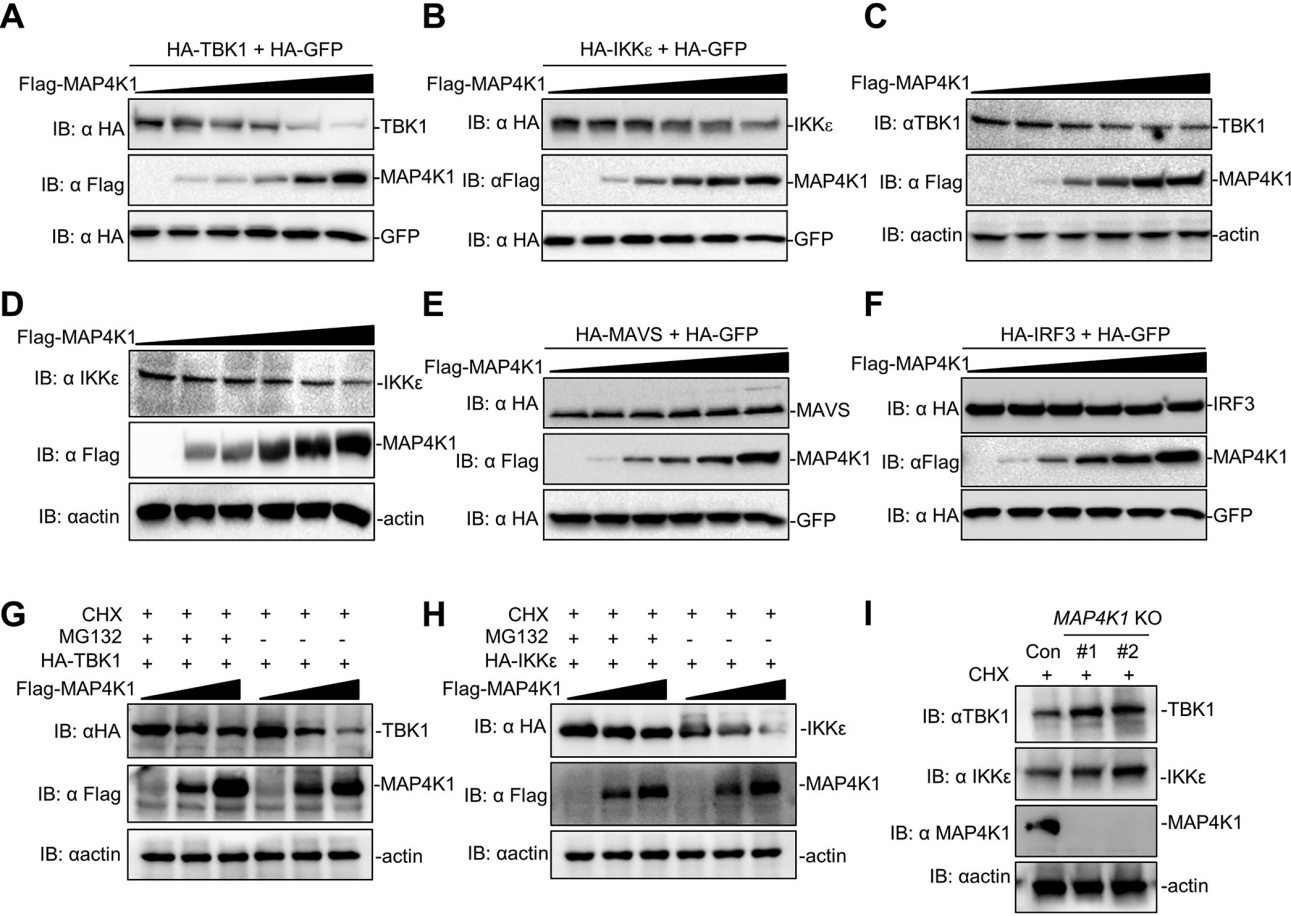
MAP4K1 promotes the degradation of TBK1/IKKε. (A, B) 293T cells (∼1 × 10^6^) were seeded in 6-well plates and transfected with HA-tagged TBK1 (2 μg) (A) or IKKε (2 μg) (B) and HA-GFP (0.5 μg) as well as increasing amounts of Flag-MAP4K1 expression vector (0, 0.1, 0.2, 0.4, 0.8, 1.6 μg). (C, D) Cells were only transfected with increasing amounts of Flag-MAP4K1 expression vector (0, 0.1, 0.2, 0.4, 0.8, 1.6 μg). Twenty hours after transfection, cells were lysed, and lysates were analyzed by immunoblotting. (E, F) Similar immunoblotting experiments were performed except cells were transfected with HA-tagged MAVS (E) and IRF3 (F). (G, H) 293T cells (∼1 × 10^6^) were seeded in 6-well plates and were transfected with HA-TBK1 (2 μg) (G) or HA-IKKε (2 μg) (H) together with increasing amounts of Flag-MAP4K1 (0, 1, 2 μg). The cells were treated with CHX (20 μM) in the presence or absence of MG132 (2 μM) for 8 h in a 37°C incubator. (I) *MAP4K1*-knockout THP1 cells and control cells were treated with CHX for 12 h in a 37°C incubator and analyzed by immunoblotting.

According to the mechanism of MAP4K1 function in lymphocyte signaling, we further examined the effects of MAP4K1 on RLR signaling molecules. As shown in the previous detection of whole cell lysate (WCL), we observed that MAP4K1 probably reduced the expression of TBK1 and IKKε under infected and uninfected condition (Fig. 1C, 2E, 4C and D). To test this, we monitored the degradation of TBK1/IKKε with increasing doses of MAP4K1. The results indicated that MAP4K1 overexpression significantly induced the degradation of TBK1 and IKKε in a dose-dependent manner (Fig. 5A and B). Consistent with the results, we examined the effects of MAP4K1 on endogenous TBK1/IKKε and found that MAP4K1 also promoted degradation of endogenous TBK1/IKKε in a dose-dependent manner (Fig. 5C and D). However, MAP4K1 overexpression had no obvious degradation effect on the adaptor MAVS and transcription factor IRF3 (Fig. 5E and F). It has been reported that TBK1 stability can be regulated by the ubiquitin-dependent proteasome pathway, which was inhibited by proteasome inhibitor MG132. To further determine whether MAP4K1 degrades TBK1/IKKε by the ubiquitin-proteasome pathway, we found that degradation of TBK1/IKKε induced by MAP4K1 was blocked by MG132 (Fig. 5G and H). In addition, endogenous TBK1/IKKε was increased in *MAP4K1*-knockout THP-1 cells with CHX/MG132 treatment (Fig. 5I). Taken together, these results demonstrated that MAP4K1 is essential for disassembly of the MAVS-associated complex by promoting the degradation of TBK1/IKKε.

### MAP4K1 enhances the polyubiquitination of TBK1/IKKε.

**FIG 6:**
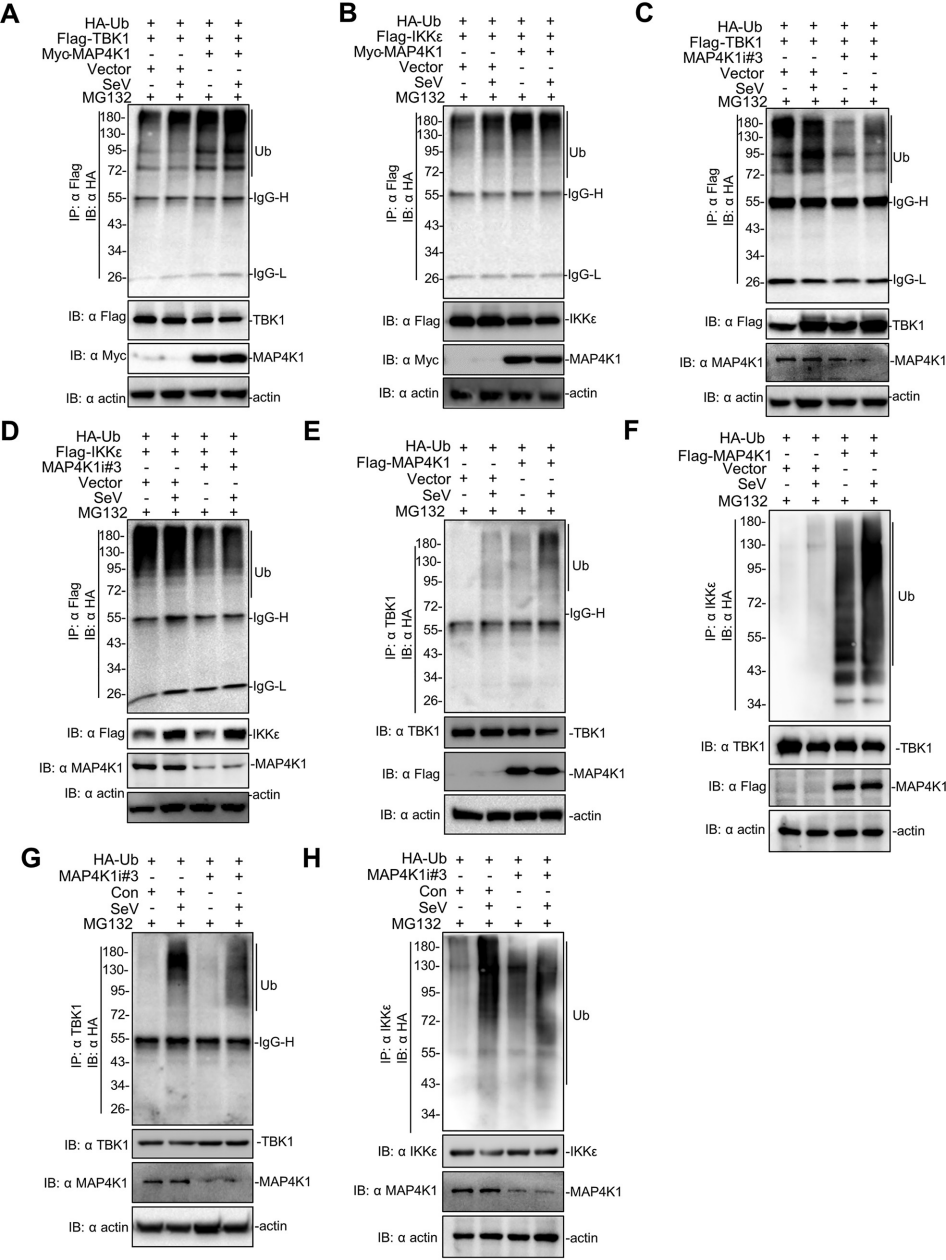
MAP4K1 enhances the polyubiquitination of TBK1/IKKε. (A) 293T cells (∼4 × 10^6^) were seeded in 100-mm dishes and were transfected with Flag-tagged TBK1 (8 μg), Myc-MAP4K1 (8 μg), and HA-ubiquitin (8 μg). After transfection for 12 h, the cells were treated with SeV or not for 10 h and MG132 for 8 h. (B) Similar immunoprecipitation and immunoblotting experiments were performed on knockdown levels by transfecting MAP4K1-specific siRNA or control (8 μg). (C, D) 293T cells were transfected with Flag-MAP4K1 (8 μg) and HA-ubiquitin (8 μg). After transfection for 12 h, the cells were treated with SeV or not for 10 h and MG132 for 8 h. Similar immunoprecipitation experiments were performed with TBK1. (E, F) Similar immunoprecipitation and immunoblotting experiments were performed except cells were transfected with Flag-IKKε. (G, H) Similar as panels C and D. The cells were harvested and lysed, followed by immunoprecipitation with anti-IKKε and immunoblotting analysis with indicated antibodies.

Consistent with the notion that MAP4K1 induced the proteasomal degradation of SLP-76 and BLNK in adaptive immunity, we assumed that MAP4K1 regulates innate immunity by influencing ubiquitination of TBK1/IKKε for proteasomal degradation. To investigate this hypothesis, ubiquitination assays were performed to examine the regulation mechanisms of MAP4K1 on the degradation of TBK1/IKKε. We transfected Flag-tagged TBK1 together with the hemagglutinin (HA)-tagged ubiquitin (HA-ub) in HEK293T cells in the presence or absence of MAP4K1. The results indicated that MAP4K1 remarkably augmented the ubiquitination of TBK1, and the level of TBK1 ubiquitination was increased more after SeV infection (Fig. 6A). MAP4K1 also enhanced the polyubiquitination of IKKε (Fig. 6B). Consistently, MAP4K1 knockdown attenuated the ubiquitination of TBK1/IKKε (Fig. 6C and D). Furthermore, similar results were obtained by detecting endogenous TBK1/IKKε ubiquitination levels and found that MAP4K1 promoted the polyubiquitination of endogenous TBK1/IKKε (Fig. 6E and F). In addition, knockdown of MAP4K1 restrained the polyubiquitination of endogenous TBK1/IKKε with SeV treatment (Fig. 6G and H). Collectively, these results suggested that MAP4K1 could regulate the activity of TBK1/IKKε by ubiquitination.

### DTX4 is responsible for MAP4K1-mediated TBK1/IKKε ubiquitination.

**FIG 7:**
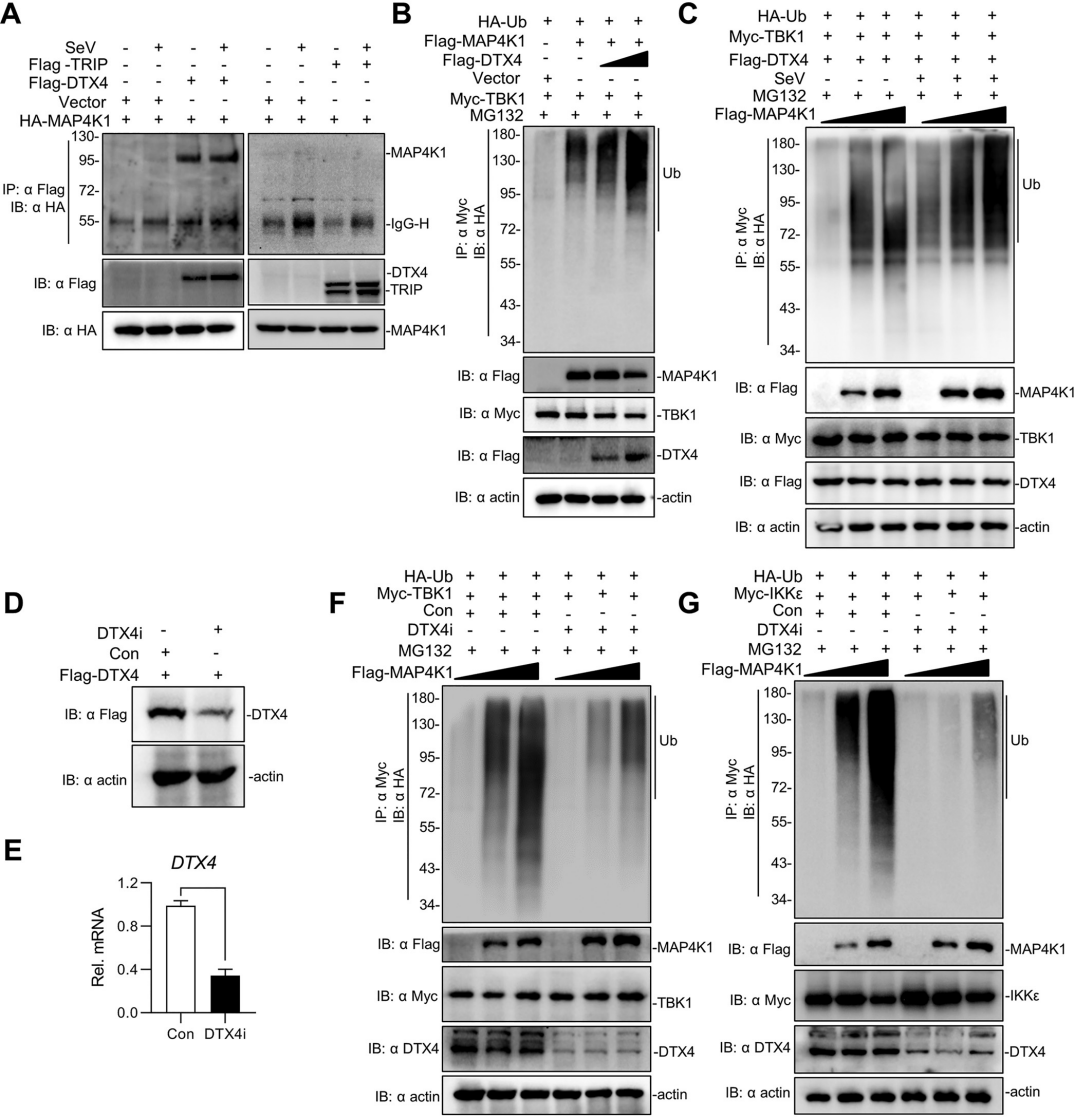
DTX4 is responsible for MAP4K1-mediated TBK1/IKKε ubiquitination. (A) 293T cells (∼4 × 10^6^) were transfected with HA-MAP4K1 and Flag-DTX4 or Flag-TRIP (8 μg). After transfection for 12 h, the cells were treated with SeV or not for 10 h. Lysates were immunoprecipitated with anti-Flag and analyzed by immunoblotting with the indicated antibodies. (B) 293T cells were transfected with Myc-tagged TBK1, HA-Ub, and Flag-MAP4K1 (5 μg) as well as increasing amounts of Flag-DTX4 expression vector (0, 2, 4 μg). After transfection for 12 h, the cells were treated with MG132 for 8 h. The cells were harvested and lysed, followed by immunoprecipitation with anti-Myc. (C) 293T cells were transfected with Myc-tagged TBK1, HA-Ub, and Flag-DTX4 (5 μg) as well as increasing amounts of Flag-MAP4K1 expression vector (0, 2, 4 μg). The cells were treated with SeV or not for 10 h and MG132 for 8 h after transfection for 12 h. Coimmunoprecipitation and immunoblotting analysis of extracts were performed with indicated antibodies. (D) Immunoblotting analysis of extracts of 293T cells transfected with DTX4 shRNA or control together with Flag-DTX4. (E) Effect of DTX4 shRNA on the transcription of DTX4. qPCR analysis of 293T cells transfected with DTX4 shRNA or control. (F, G) Immunoprecipitation and immunoblotting analysis of 293T cells transfected with Myc-TBK1 or IKKε, HA-Ub, DTX4 shRNA or control (5 μg) together with increasing amounts of Flag-MAP4K1 (0, 2, 4 μg) and treated with MG132 as above.

Although MAP4K1 is not an E3 ubiquitin ligase, it always affects the stability of target proteins (e.g., SLP-76, BLNK) by acting as an adaptor to recruit an E3 ubiquitin ligase. Therefore, we inferred that MAP4K1 might bind an E3 ubiquitin ligase to affect TBK1 ubiquitin. It has been documented that TBK1 undergoes degradation by E3 ubiquitin ligase DTX4 or TRIP (19, 34). Therefore, we reasoned that MAP4K1 works together with DTX4 or TRIP to regulate the level of TBK1 ubiquitination. To verify this hypothesis, we performed immunoprecipitation and immunoblotting analyses in 293T cells by transfecting HA-MAP4K1 and Flag-tagged DTX4 or TRIP. As a result, we found that MAP4K1 interacted with DTX4 but not with TRIP (Fig. 7A). Furthermore, MAP4K1 enhanced ubiquitination of TBK1 in a dose-dependent manner of DTX4 (Fig. 7B). Consistent with this observation, MAP4K1 enhanced ubiquitination of TBK1 relative to ubiquitination levels in the presence of DTX4 expression alone. DTX4 affected the ubiquitination of TBK1 in a dose-dependent manner of MAP4K1 (Fig. 7C).

To demonstrate the involvement of DTX4 in MAP4K1-mediated TBK1 ubiquitination, we knocked down the expression of endogenous MAP4K1 by DTX4-specific lentivirus short hairpin RNA (shRNA) as previously reported. We first tested the knockdown efficiency of DTX4 shRNA by immunoblotting analysis and found that the expression of DTX4 was inhibited by DTX4 shRNA (Fig. 7D and E). Knockdown of endogenous DTX4 markedly abrogated the promotion of TBK1 ubiquitination by MAP4K1 (Fig. 7F). Similarly, knockdown of endogenous DTX4 also abrogated the effect of MAP4K1 on IKKε ubiquitination (Fig. 7G). These results suggested that DTX4 has a significant role in the ubiquitination of TBK1/IKKε in a MAP4K1-dependent manner.

### MAP4K1 promotes K48-linked ubiquitination of TBK1/IKKε.

**FIG 8:**
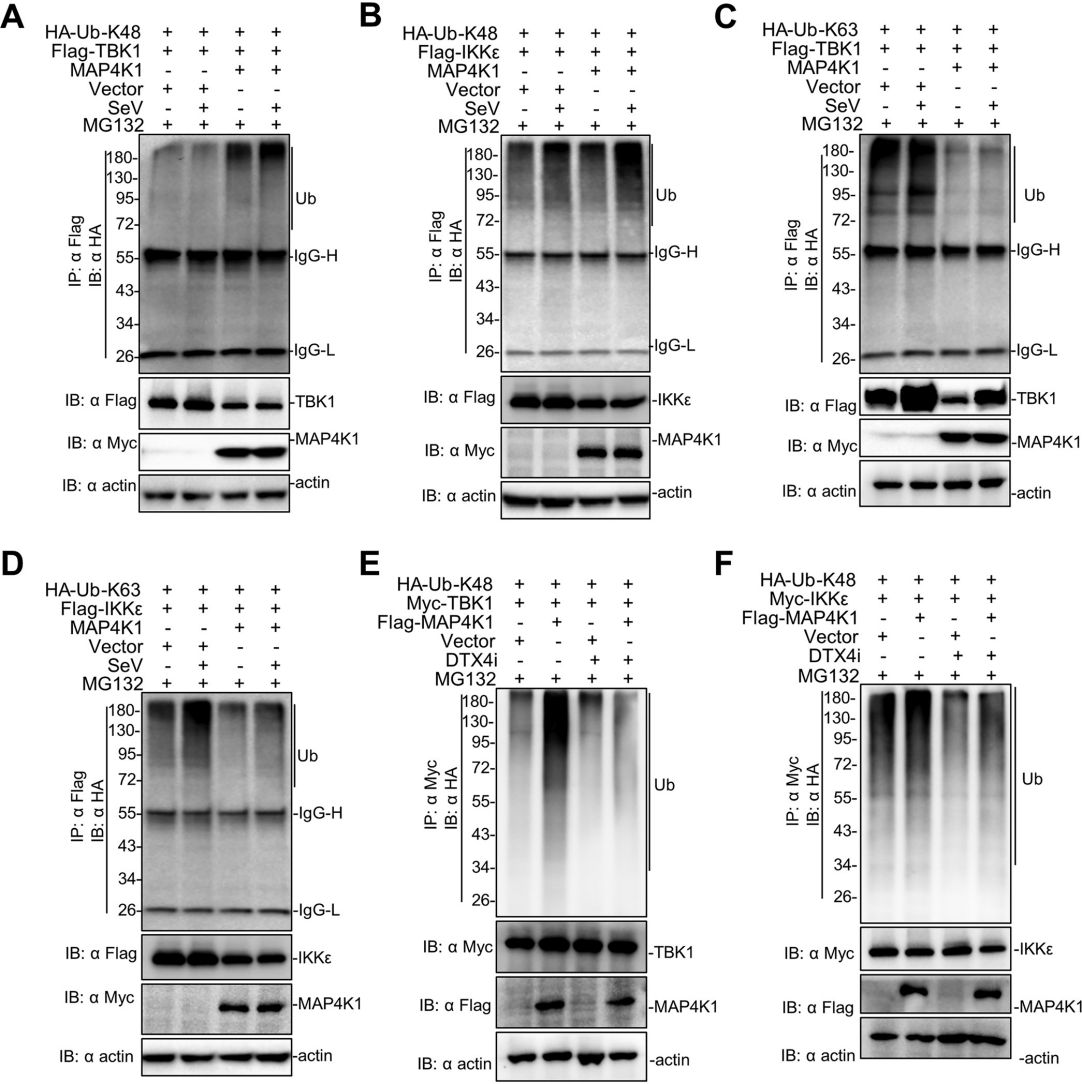
Identification of the type of ubiquitination for TBK1/IKKε. (A to D) 293T cells (∼4 × 10^6^) were transfected with Flag-tagged TBK1 or IKKε (8 μg) and Myc-MAP4K1 (8 μg) together with HA-tagged K48 (A, B) or K63 ubiquitin (8 μg) (C, D). After transfection for 12 h, the cells were treated with SeV or not for 10 h and MG132 for 8 h. Ubiquitination levels were detected by the method described above and immunoblotting analysis with indicated antibodies. (E, F) 293T cells were transfected with Myc-tagged TBK1 (E) or IKKε (F), HA-tagged K48 ubiquitin, and Flag-MAP4K1 together with DTX4 shRNA or control. The cells were treated with MG132 for 8 h. Coimmunoprecipitation and immunoblotting analyses of extracts were performed with indicated antibodies.

It has been reported that viral infection induces K48-linked and K63-linked ubiquitination of TBK1, which is essential to the activation of IFN-I signaling (19, 35). To determine the type of TBK1 ubiquitination in the following experiments, we introduced two HA-tagged ubiquitin mutants, K48, and K63 ubiquitin, where all lysine (Lys) residues were replaced by arginine (Arg) residues except for at site 48 or 63, respectively (36). We added MG132 to 293T cells to block protein degradation after SeV infection. The results showed that MAP4K1 significantly promoted K48-linked ubiquitination of TBK1/IKKε (Fig. 8A and B). In the same experiments, we found that MAP4K1 may have a much weaker effect on K63-linked ubiquitination of TBK1/IKKε (Fig. 8C and D). To verify the role of DTX4 in the ubiquitination of TBK1, we assessed K48-linked ubiquitination of TBK1 in cells transfected with DTX4-specific shRNA. In cells transfected with control shRNA, we found that MAP4K1 promoted K48-linked polyubiquitination of TBK1; however, the promoting effect of MAP4K1 on the K48-linked polyubiquitination of TBK1 was completely abolished when DTX4 was knocked down (Fig. 8E). Similarly, the K48-linked polyubiquitination of IKKε was also significantly attenuated in cells transfected with MAP4K1 and DTX4-specific shRNA (Fig. 8F). These results suggested that MAP4K1 recruits DTX4 to interact with TBK1 to promote the K48-linked polyubiquitination of TBK1/IKKε.

**FIG 9:**
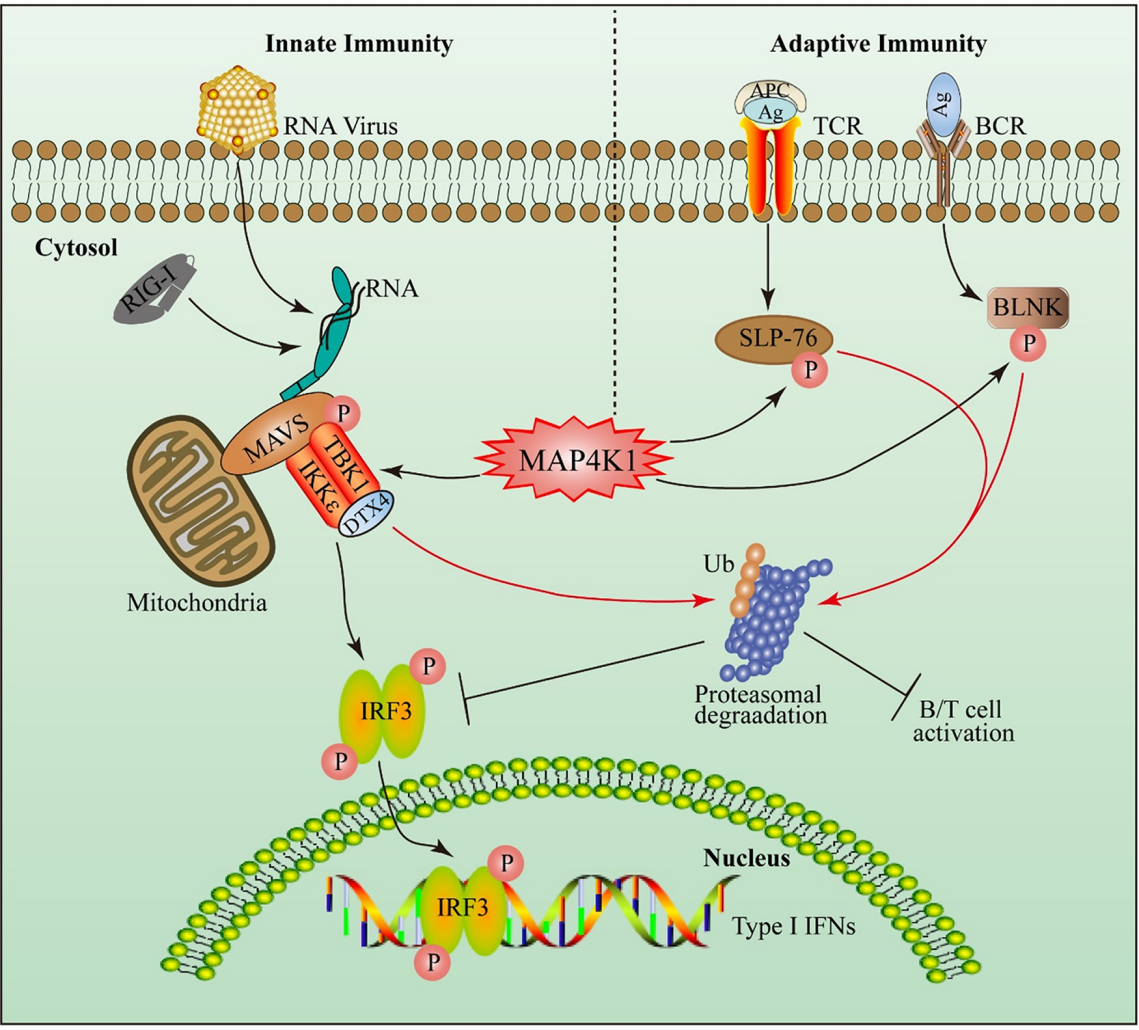
A model for the regulation of MAP4K1 in RLR signaling and lymphocyte signaling. MAP4K1 has a critical role in RLR signaling and lymphocyte signaling. In lymphocyte signaling, following activation of TCR or BCR signaling, MAP4K1 was activated by recruiting the adaptor SLP-76 or BLNK, and the activated MAP4K1 feedback phosphorylated the corresponding adaptor. Then, the phosphorylated adaptors were degraded by UPS and inhibited T/B cell activation. In RLR signaling, following invasion by an RNA virus, RIG-I detected the RNA of the virus and was recruited to the adaptor MAVS, which formed a signalosome complex. Also, MAP4K1 was recruited to the MAVS signalosome and induced proteasomal degradation of TBK1/IKKε. MAP4K1 triggered the disassembly of the MAVS signalosome and inhibited the activation of IRF3 and subsequently repressed the production of IFN-I.

Taken together, we found that MAP4K1 negatively regulated antiviral innate immunity by targeting TBK1/IKKε for degradation via the ubiquitin ligase DTX4. Our study identified MAP4K1 as a novel TBK1 inhibitor and elucidated the mechanism of MAP4K1 as a negative regulator of innate antiviral immunity, which provided a link between innate immunity and adaptive immunity with the function of MAP4K1. A schematic model is shown in Fig. 9.

## DISCUSSION

TBK1 functions as a node protein that can receive upstream inputs and modulate downstream outputs in multiple signaling pathways, including inflammation and innate immune response (7). IFN-I must be strictly and accurately regulated to avoid autoimmune disorders and self-damage. Therefore, TBK1 has been proposed to be a potential target, and its inhibitors may be used in clinical therapy for autoimmune diseases (21). It has been reported that TBK1 activity can be regulated by kinase activity modulation, posttranslational modification such as phosphorylation or ubiquitination, as well as prevention of functional TBK1-containing complex formation (37, 38). It was reported that NLRP4-DTX4, TRIP Siglec1-TRIM27, THOC7, and TRAF3IP3 promote K48-linked polyubiquitination of TBK1 (19, 34, 39, 40). Although many inhibitors were found or patented, there is still much to be understood by searching more interaction partners of TBK1.

In this study, we reported MAP4K1 as a novel interacting partner of TBK1 and elucidated the mechanism in antiviral innate immunity. Overexpression and knockdown of MAP4K1 suggested that MAP4K1 significantly inhibited the activation of SeV-triggered IFN-β, ISRE, and NF-κB promoters in a dose-dependent manner. Additionally, MAP4K1 promoted the degradation of TBK1/IKKε by increasing K48-associated polyubiquitination. Together, our results suggested that MAP4K1 negatively regulates antiviral innate immunity by targeting TBK1/IKKε for degradation.

MAP4K1 is a Ste20-related serine/threonine kinase containing a kinase domain in the N-terminus and a regulatory domain (named citron homology domain) in the C-terminus (41). It has been reported that MAP4K1 is activated by multiple stimuli, including inflammatory cytokines and antigen receptor triggering, and participates in multiple signaling pathways, including AP-1, NF-κB, JNK, and apoptosis in lymphocytes (22, 42). Activity of MAP4K1 can be modulated by the regulatory domain, which can separate the kinase domain by caspase-3 activity (43). Therefore, MAP4K1 has different functional roles in different signaling pathways or cell types. MAP4K1 negatively regulates ERK and AP-1 in T cell lines, whereas it has no effect on ERK in B cell lines (44, 45). MAP4K1 exhibits dual functions as a positive and negative regulator in the TCR-mediated NF-κB signaling pathway (41, 45, 46). Consistent with this idea, our results showed that MAP4K1 attenuated the activation of NF-κB induced by SeV, while possibly enhancing NF-κB activity without treatment SeV (Fig. 1B). However, no literature we found has shown that MAP4K1was involved in antiviral innate immunity.

As shown in Fig. 9, MAP4K1 acts as a negative regulator in lymphocyte signaling and the RLR antiviral pathway. MAP4K1 phosphorylated the adaptor SLP-76 and BLNK following TCR and BCR stimulation and induced the corresponding adaptor’s degradation by ubiquitination. Inspired by this, we first detected the effect of MAP4K1 on the adaptor MAVS protein stability. However, MAP4K1 had little or no obvious effect on MAVS degradation. Interestingly, we observed that MAP4K1 possibly enhances MAVS phosphorylation in a dose-dependent pattern (Fig. 5D; very weak but specific band). Additional research is merited to clarify the function of MAP4K1 on MAVS. For the role of MAP4K1 in the RLR antiviral pathway, a possible explanation is that MAP4K1 regulated the RLR antiviral pathway mainly at the TBK1/IKKε level and promoted the disassembly of the formation of the MAVS signalosome by inducing proteasomal degradation of TBK1/IKKε but not MAVS. Meanwhile, we found that MAP4K1 promotes virus replication in the cell by measuring virus genomic RNA (Fig. S3 in the supplemental material). Therefore, MAP4K1 attenuated IFN-β production and antiviral responses.

It has been reported that MAP4K1 is a functional component of the endogenous canonical IKK complex and regulated NF-κB activity by the mechanism that full-length MAP4K1 enhances IKKβ phosphorylation, and cleaved MAP4K1 binds to IKKα with IKKβ and suppresses NF-κB activity (41). Our report is the first to identify the vital role of MAP4K1 in RLR antiviral responses and how MAP4K1 affects the activation of noncanonical IKKs, including TBK1with IKKε by degradation. Therefore, MAP4K1 is a pivotal regulator of IKK activity, and more studies are needed to characterize its underlying mechanism in innate immunity.

## MATERIALS AND METHODS

### Cell culture, virus, and reagents.

HEK293T cells were grown in high-glucose Dulbecco’s modified Eagle’s medium (DMEM; Gibco, Grand Island, NY, USA) containing 10% fetal bovine serum (FBS; Gibco), penicillin (100 U/mL; Solarbio), and streptomycin (100 mg/mL; Solarbio). THP1 cells were grown in RPMI 1640 medium with 10% FBS at 37°C in a 5% CO_2_ incubator. Sendai virus (SeV) was provided by Hong-Bing Shu (Wuhan University, China). The mouse monoclonal antibodies specific for HA or Flag were obtained from Sigma-Aldrich (Darmstadt, Germany). Mouse antibodies against IRF3 (1:2,000; sc-33641, Santa Cruz Biotechnology Inc.), MAP4K1 (1:2,000; sc-374183, Santa Cruz Biotechnology Inc.), and actin (1:5,000; BM0005, Boster Biological Technology) and rabbit antibodies against Myc (1:4,000; AE070, ABclonal Technology), RIG-I signaling pathway proteins (1:2,000; RIG-I pathway antibody sampler kit, 8348, Cell Signaling Technology), including TBK1 and phospho-TBK1 (Ser172) with IRF3 (Ser396), and rabbit antibodies against NF-κB signaling pathway proteins (1:2,000; NF-κB pathway sampler kit, 9936, Cell Signaling Technology), including p65 with phospho-p65 (Ser536), were purchased from the indicated companies. Pure nitrocellulose blotting membrane was purchased from Pall Corporation (NY, USA). The dual-luciferase reporter assay system kit (E1980), total RNA extraction kit (LS1040), reverse transcription system kit (LS2050), and a SYBR quantitative PCR kit (qPCR master mix kit, LS2062) were purchased from Promega (Madison, WI, USA).

### Constructs and RNAi.

Mammalian expression vectors for human Flag- or HA-tagged RIG-I, RIG-I-N (containing only the N-terminal CARD domain, 1 to 284 amino acids), MAVS, TBK1, IKKε, IRF3 and its mutant IRF3-5D (the phosphorylation sites as Ser396, Ser398, Ser402, Thr404, and Ser405 residues were replaced by aspartic acid in the C-terminal end of IRF-3) (47), ubiquitin and its mutants K48 and K63 ubiquitin, IFN-β promoter luciferase reporter, ISRE, and NF-κB luciferase reporter constructs were described previously (10, 39, 48). The full-length human *MAP4K1* was cloned into mammalian expression vector pRK5 with an N-terminal HA or Flag tag and pcDNA3.1 with an N-terminal Myc tag by standard molecular biology techniques. *MAP4K1*-specific siRNA constructs were generated by the pSuper.retro vector (OligoEngine, Seattle, WA, USA) with double-strand oligonucleotides targeting *MAP4K1* sequences using protocols recommended by the manufacturer. The following sequences were designed for targeting human *MAP4K1* mRNA: *MAP4K1*-RNAi 1, 5′-CCTGGATCTTCTTGACAAA-3′; *MAP4K1* RNAi 2, 5′-CCTGATCCTGGATCTTCTT-3′, and *MAP4K1* RNAi 3, 5′-CCAGACGCCTCTCTTTCAT-3′. The following shRNA sequences were used: control shRNA sequence (5′-CAACAAGATGAAGAGCACCAA-3′) and *Dtx4* shRNA (5′-TTAAGGCAGCCGTGGTCAATG-3′).

### Dual-luciferase reporter assay.

HEK293T cells (∼2.5 × 10^5^) were seeded in 24-well plates and transiently cotransfected with a firefly luciferase reporter gene (IFN-β promoter, ISRE, or NF-κB luciferase reporter; 100 ng/well) and *Renilla* luciferase plasmid (pRL-TK; 50 ng/well) as a control together with indicated plasmids or vector. Cells were treated with SeV or not for 10 h after transfection for 12 h and collected and lysed with 1× passive lysis buffer (PLB) as per the manufacturer’s protocols. According to the manufacturer’s protocol, cellular extracts were used for luciferase assay by a dual-luciferase reporter assay system kit with a GloMax 20/20 luminometer (Promega). Relative luciferase activity was obtained by the ratio of firefly luciferase activity to *Renilla* luciferase activity, and the data are expressed as the mean ± standard error of the mean (SEM) of three repeated experiments.

### Immunoprecipitation, immunoblotting, and dimerization assay.

The 293T cells (∼4 × 10^6^) were plated in 100-mm dishes and transfected with indicated plasmids. Twenty hours after transfection, cells were lysed with Triton lysis buffer (1% Triton, 20 mM Tris-HCl pH 7.5, 150 mM NaCl, 1 mM EDTA, 1 mM phenylmethylsulfonyl fluoride (PMSF), 5 mM dithiothreitol (DTT), 10 μg/mL aprotinin, and 10 μg/mL leupeptin). The cellular lysates were prepared by centrifugation at 12,000 rpm for 10 min at 4°C. For each immunoprecipitation, ∼30 μL of protein G/A-Sepharose beads (GE Healthcare, Piscataway, NJ) was incubated with cellular lysates and appropriate amounts of indicated antibodies overnight at 4°C and then washed with 1 mL of lysis buffer containing 1 M NaCl for 15 min and 1 mL of lysis buffer without NaCl two times. The proteins were subsequently separated by SDS-PAGE as previously described (39, 49). For the IRF3 dimerization assay, cells were treated with SeV or not for indicated time points and then collected and lysed by lysis buffer. Cellular extracts were dissolved in native PAGE sample buffer (62.5 mM Tris-Cl [pH 6.8], 15% glycerol, and 1% deoxycholate) and analyzed by native PAGE (50, 51).

### CRISPR-Cas9.

The small guide RNAs (sgRNAs) to human *MAP4K1* were taken from a lentiCRISPR sgRNA library designed by Feng Zhang (52). The method of genome engineering and lentivirus production was previously described (53, 54). Briefly, the vesicular stomatitis virus G protein (VSV-G) pseudotyped lentivirus was produced in HEK293T cells and concentrated by lentivirus concentration reagent (Biomega, San Diego, USA). The visible virus-containing pellet was obtained by centrifuging at 3,000 rpm for 30 min at 4°C and resuspended in 1 mL of RPMI 1640 medium. Two days after infection, the THP1 cells were placed under 1 μg/mL puromycin selection for 10 days. The single clone cells were obtained by a limited dilution method in 96-well plates. The following sgRNA sequences were used: *MAP4K1* sg 1 (5′-CCTTTGCAAGGCTCGAGACA-3′) and *MAP4K1* sg 2 (5′-CACGTATGGGGAAGTCTTTA-3′).

### Real-time fluorescent quantitative PCR analyses.

Total RNAs of 293T cells transfected with the indicated plasmids were prepared using an RNA-extraction kit (Promega) as per the manufacturer’s instructions. RNAs (1 μg) were transcribed into cDNA by a reverse transcription kit (Promega). Real-time fluorescent quantitative PCR analyses were performed with a SYBR quantitative PCR kit (Promega). The relative mRNA levels of target genes in the samples were assessed by the comparative *C_T_* method and were normalized by β-actin. The primers of the target genes were as follows: *ACTB* gene forward (5′-GTCGTCGACAACGGCTCCGGCATG-3′), *ACTB* gene reverse (5′-ATTGTAGAAGGTGTGGTGCCAGAT-3′), *MAP4K1*gene forward (5′-CGCCTACTGGCAAGGAAGAA-3′), *MAP4K1* gene reverse (5′-TGTGGAAGAGCACCGACTTC-3′), *IFNB1* gene forward (5′-CTAACTGCAACCTTTCGAAGC-3′), *IFNB1* gene reverse (5′-GGAAAGAGCTGTAGTGGAGAAG-3′), *CXCL10* gene forward (5′-GGTGAGAAGAGATGTCTGAATCC-3′), *CXCL10* gene reverse (5′-GTCCATCCTTGGAAGCACTGCA-3′), *RANTES* gene forward (5′-GGCAGCCCTCGCTGTCATCC-3′), *RANTES* gene reverse (5′-GCAGCAGGGTGTGGTGTCCG-3′), *ISG56* gene forward (5′-TCATCAGGTCAAGGATAGTC-3′), and *ISG56* gene reverse (5′-CCACACTGTATTTGGTGTCTAGG-3′).

### Statistical analysis.

All statistical analyses were performed using GraphPad Prism 8.0, and values are represented as the mean ± SEM of three independent experiments. Statistical significance of differences between two groups was determined using an unpaired Student’s *t* test, and those of differences among multiple groups (*n* ≥ 3) were analyzed by two-way analysis of Sidak’s multiple comparison *post hoc* analysis. Asterisks indicate statistical significance (*, *P < *0.05; **, *P < *0.01; ***, *P < *0.001).
