# Supplementary material for: The Kinase MAP4K1 Inhibits Cytosolic RNA-Induced Antiviral Signaling by Promoting Proteasomal Degradation of TBK1/IKKε
Source: Microbiol Spectr. 2021 Dec 15;9(3):e01458-21. doi: 10.1128/Spectrum.01458-21 (PMC8672915; doi:10.1128/Spectrum.01458-21)
Supplement: SUPPLEMENTAL FILE 1 — Supplemental material. Download SPECTRUM01458-21_Supp_1_seq13.pdf, PDF file, 0.3 MB [file spectrum01458-21_supp_1_seq13.pdf]

Supplementary Information for

The Kinase MAP4K1 Inhibits Cytosolic RNA induced anti-viral signaling by Promoting Proteasomal Degradation of TBK1/IKKε

Tian-Sheng He, Jingping Huang, Tian Chen, Zhi Zhang, Cai Kuntai, Yu Jingge, Liang-Guo Xu[1¶]

[¶]Address correspondence to:

Liang-Guo Xu, email: xul@jxnu.edu.cn

This PDF file includes:

Figures S1 to S3

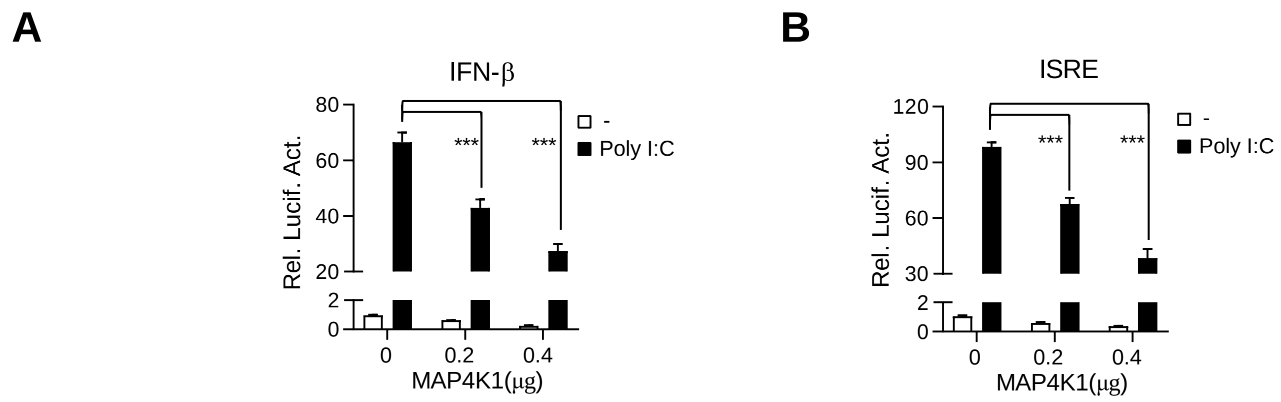

Fig. S1 MAP4K1 negatively regulates RLR signaling pathway

(A-B) MAP4K1 inhibited the activation of the IFNB1 and ISRE induced by PolyI:C. Similar luciferase assay was performed as Fig. 1B except that PolyI:C (0.2 μg) were transfected or not for 10 h.

He et al., Fig. S2

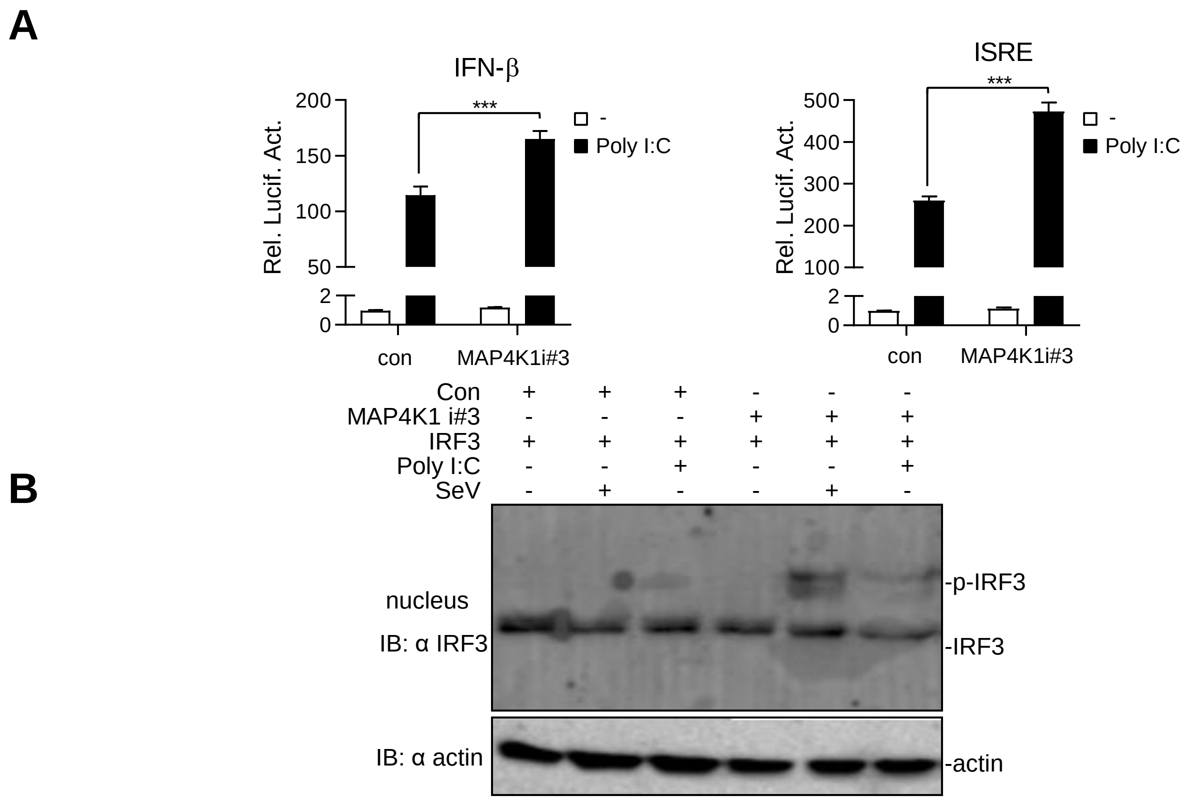

Fig. S2 Knockdown of MAP4K1 potentiates antiviral responses

(A) Knockdown of MAP4K1 potentiated the activation of the IFNB1 and ISRE induced by PolyI:C. Similar luciferase assay was performed as Fig. 2C except that PolyI:C (0.2μg) were transfected or not for 10 h. (B) Knockdown of MAP4K1 enhanced the phosphorylation of IRF3. 293T cells transfected MAP4K1 RNAi#3 or control and IRF3, and treated with SeV or transfected PolyI:C for 10 h. Nuclear protein was extracted and immunoblotting analyzed with IRF3.

He et al., Fig. S3

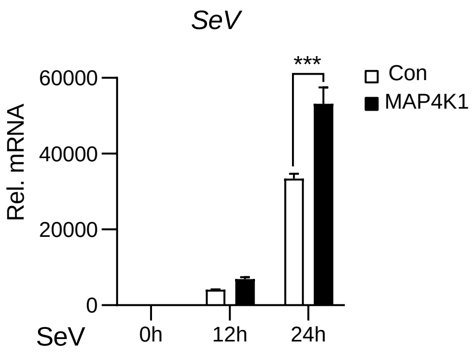

Fig. S3 MAP4K1 promotes SeV replication.

HEK293T cells were transfected with MAP4K1 or control for 12 h followed by SeV infection for the for 1 h, and then cells were replaced with new medium for another indicated time. SeV genomic RNA in cells was extracted for realtime PCR tests by specific genomic primers.
